# Development and Validation of a Protective Behavioral Strategies Scale for Individuals who use Opioids: Preliminary Findings and Future Directions

**DOI:** 10.21203/rs.3.rs-3467052/v1

**Published:** 2023-10-23

**Authors:** Rachel Luba, Manesh Gopaldas, Suky Martinez, Sandra D. Comer

**Affiliations:** Columbia University Irving Medical Center; Columbia University Irving Medical Center; Columbia University Irving Medical Center; Columbia University Irving Medical Center

**Keywords:** Opioid Use, Protective Behavioral Strategies, Harm Reduction, Factor Analysis

## Abstract

**Background:**

Protective Behavioral Strategies (PBS) are individually implemented harm reduction (HR) strategies to reduce the frequency or severity of risks associated with drug use. Existing scales measuring PBS for alcohol and cannabis suggest PBS are associated with reductions in associated problems. Despite many HR strategies related to opioid use, no PBS scale has been developed in the context of opioid use. To address this gap, this study aimed to test and validate a PBS scale for individuals using opioids (PBSO).

**Methods:**

An online survey utilized a 32-item PBS scale for individuals endorsing recent opioid use, and measured opioid use frequency, HR service use, and experience of opioid overdose. PBSO items were rated on a Likert scale ranging from “never” (0) to “always” (6), and an exploratory factor analysis (EFA) examined factor structure.

**Results:**

In the current sample (n=499; 32% female), EFA suggested a 3-factor structure among the 28 items retained, accounting for 51% of total variance. Factor 1 reflected health-service seeking, Factor 2 reflected individually-implemented and dose-reduction strategies, Factor 3 reflected social strategies, and Factor 4 reflected strategies related to injection drug use. Endorsement of PBSO items were slightly above “occasional” (3). PBSO use appeared positively related to past-month HR service utilization and negatively related to opioid use frequency.

**Conclusions:**

Findings provide preliminary support for the PBSO scale as a valid and reliable measure. Further work is needed to test this scale in larger samples, and future work should explore the association between PBSO and relevant health outcomes, and whether factor scores differentially impact these outcomes.

## 1. Introduction

The U.S. opioid overdose (OD) crisis has reached historic levels and continues to evolve. An estimated 79,117 Americans died from an opioid-related OD in the first nine months of 2022 (CDC Wonder, 2023), and approximately 2.7 million Americans meet criteria for opioid use disorder (OUD) each year (CDC/NCHS, 2022_a_; CDC/NCHS, 2022_b_). Medications for OUD (MOUD) protect against overdose, reduce opioid use, and improve health outcomes; however, only a fraction of those with OUD access, initiate, or remain engaged in treatment, with some estimates suggesting that nearly 90% of those who may benefit from medication for OUD (MOUD) do not receive it (Jones & McCance-Katz, 2019; Krawczyk, 2022; Mancher et al., 2019; Simpson et al., 2022). With an increasingly toxic drug supply containing fentanyl and xylazine, (Ciccarone, 2021; Friedman & Hansen, 2022; Johnson et al., 2021) and a majority of people with OUD unable to access or not engaged with MOUD (Krawczyk, 2022), an increased focus is needed on harm reduction (HR) service implementation for those with OUD and other substance use disorders.

Broadly, HR provides tools, resources, and strategies for individuals who use drugs that can help mitigate risks associated with drug use, regardless of an individual’s interest in or engagement with formal treatment (Single, 1995; National Harm Reduction Coalition [NHRC], 2020). In the context of OUD, common HR strategies include syringe access, overdose education and naloxone distribution (OEND), access to testing for infectious diseases, and more recently in the U.S., overdose prevention centers (OPCs). Research supports the role of HR in reducing OD deaths and providing a platform for connecting individuals with vital health and social resources, as well as offering linkage to treatment for those who are interested (Nassau et el., 2022; Puzhko et al, 2022; Ritter & Cameron, 2006; Ruiz et al., 2019; Surratt et al., 2020). The 2022 National Drug Control Strategy (Office of National Drug Control Strategy, 2022) prioritized HR for the first time in U.S. history, and with increased enthusiasm around HR strategies, research can help clarify the precise strategies people are using – and have access to – to stay alive. Development and use of a simple scale to measure HR utilization may allow researchers to develop insights and tools to implement these strategies and inform straightforward educational or behavioral interventions as the opioid epidemic continues to evolve.

Protective Behavioral Strategies (PBS) are broadly defined as individually implemented HR strategies aimed at reducing the frequency or severity of risks associated with drug use. To date, most research on PBS has focused on alcohol and cannabis. Previous work suggests that greater use of PBS is associated with reduced risk for alcohol and cannabis-associated problems (Peterson et al., 2021), and that PBS may attenuate other risk factors such as impulsivity and coping motives (Bravo et al., 2017). Efforts to validate prior PBS measures for alcohol and cannabis have generally provided psychometric validation of these scales, but there is some variability in the number of factors extracted using exploratory and confirmatory factor analyses (Peterson et al., 2021). Broadly, prior factor analyses have suggested underlying PBS factors relating to quantity of use, context of use, and for alcohol, “serious harm reduction” efforts (Martens et al., 2005; Mian et al., 2021; Richards et al., 2018; see Peterson et al., 2021 for review). Despite the many HR strategies relevant to opioid use, no scales have been developed to date to specifically measure PBS in the context of opioid use or explore how use of PBS may be protective. Most work has focused on evaluating the effectiveness of one or two strategies on their own (Peiper et al., 2019), rather than understanding PBS as a broader construct among those with OUD. Understanding how PBS play a role in OUD severity or associated clinical outcomes, and whether specific PBS are particularly helpful or tend to covary, may provide a low-threshold, simple, and effective tool for identifying those most at risk of adverse events associated with opioid use, and fostering responsive HR interventions. In this study, we sought to develop, administer, and validate a 32-item PBS questionnaire for individuals who endorse recent opioid or MOUD use.

## 2. Materials and Methods

Participants were recruited for a one-time web-based survey via Reddit, Bluelight, and flyers posted in the New York State Psychiatric Institute (NYSPI). An initial pool of participants was recruited with an incentive of entering a raffle to win one of eight $50 gift cards. A second pool of participants was recruited with an incentive of entering a raffle to win one of ninety-five $10 gift cards. All study procedures and survey questionnaires were approved by the NYSPI Institutional Review Board. After providing consent to participate, participants were queried about past-month use of PBS, patterns of opioid use, and past-month HR service utilization. Prior to initiating the survey, participants were asked to confirm that they were at least 18 years old and had used opioids or MOUD within the last 3 months. Participants who completed the survey were provided an option to be redirected to a separate survey to enter their email address for raffle entry and gift card eligibility. Survey responses were therefore collected in an entirely separate survey than potentially identifying information (email address).

### 2. 1 Measures

The Protective Behavioral Strategies Scale (Martens et al., 2005) was adapted for opioid use in the current survey, resulting in a 32-item PBS for Opioids (PBSO) scale. Items for the PBSO were selected based on prior versions of the PBS for alcohol and cannabis, and a review of HR strategies common among those with OUD and/or injection drug use (IDU) by authors RL, SM, MG, and SD. Authors reviewed published literature on HR for OUD (Nassau et al., 2022; Puzhko et al., 2022), as well as guidelines published by organizations such as the National Harm Reduction Coalition (NHRC; NHRC, 2020), Faces and Voices of Recovery (2019), Health and Human Services (HHS, 2022) Substance Abuse and Mental Health Services Administration (SAMHSA, 2023), and the National Institute on Drug Abuse (NIDA, 2022). In reviewing these sources, several themes emerged that were used in developing items specific to OUD or adopting items used by prior, validated PBS scales. Specifically, items focused on overdose prevention training and access to naloxone, education and resources focused on IDU and infectious diseases, syringe exchange and safe consumption strategies, utilization of MOUD, use of fentanyl test strips, and social or dose-control strategies observed among individuals with OUD (CITE). Strategies adapted directly from other validated PBS scales included purchasing opioids or drugs from a trusted source, avoiding mixing opioids with other drugs, avoiding driving while intoxicated, and using a small amount before using more. As with prior versions of the PBS, participants were instructed to rate past-month engagement in each behavior on a 6-point Likert scale ranging from never (0) to always (6), or to select N/A for items not relevant or applicable (e.g., items about IDU for those who don’t use drugs intravenously). The PBSO also included one attention-check question, on which participants were instructed to select “occasionally (3)”. For a full list of items included in the PBSO, see Table 2. One open-ended question followed presentation of the PBSO to allow participants to share additional strategies they did not feel were captured by the scale.

#### Demographic Data.

Participants were asked to provide their age, race, ethnicity, gender identity, and state in which they resided.

#### Opioid Use.

Participants were asked to describe current opioid use with emphasis on route of administration, frequency of use (days per week), typical amount of money spent on opioids per occasion (in U.S. dollars), experience of opioid overdose (OD), and connection with a treatment provider.

#### Past-Month HR Service Utilization.

Participants were presented with a list of 11 common HR services and asked to select which they had accessed in the past month. Items included: obtaining naloxone, attending a naloxone training, sterile injection supplies such as syringes, safe smoking supplies, wound care, screening for HIV and Hepatitis C infections, use at an overdose prevention center, accessing pre-exposure prophylaxis (PrEP) or post-exposure prophylaxis (PEP), and accessing fentanyl test strips. Additionally, an open-ended question was included to capture any past-month HR service utilization not listed above.

### 2.2 Statistical Analyses

Data analyses were conducted in RStudio 2023.3.0. Means and standard deviations were calculated for all quantitative variables, and frequencies and percentages were calculated for categorical variables. Internal consistency of the PBSO was assessed using Cronbach’s alpha and split-half reliability estimates. Bivariate correlations and linear regression were used to assess associations between PBSO scores and opioid use, opioid OD, and past-month HR service utilization. Prior to running the initial exploratory factor analysis (EFA), Bartlett’s test of sphericity and the Kaiser-Meyer-Olkin test were used to confirm appropriateness for EFA. An initial EFA was conducted to determine which (if any) items should be dropped and a final EFA was conducted with the set of remaining items. Based on prior recommendations for EFA (Tabachnick and Fidell, 2007), items with low loadings (< 0.32) or relatively strong loadings (≥ 0.32) on two or more factors (i.e., cross-loadings) were dropped from further analyses.

## 3. Results

A total of 1,056 individuals initiated/opened the survey with 293 participants not responding to a single question after the survey consent page. An additional 256 participants provided invalid responses, or responses in a language other than English, and were therefore not included in the present analyses. An additional eight participants incorrectly responded to the attention-check question and were removed from analyses. Therefore, a total of 499 valid responses were included in the present analysis.

Demographic characteristics are displayed in Table 1. On average, participants were aged 31.1 (SD = 8.0) years, and most were white (70%, n = 344) and non-Hispanic (70%; n = 294). With regard to gender identity, 68% of the sample (n = 327) identified as male, 28% (n = 134) identified as female, 2% (n = 10) identified as transgender, 1% (n = 5) identified as non-binary, and > 1% (n = 3) identified as genderqueer/gender-fluid. An additional 1% of participants (n = 5) stated that they did not wish to provide an answer or felt the above categories did not capture their gender identity.

Participants endorsed using opioids an average of 3.9 (SD = 2.0) days per week, with 11% of participants (n = 51) only endorsing prescribed MOUD use. Of the remaining 411 participants endorsing heroin, fentanyl, or non-prescribed opioid use, 33% (n = 152) endorsed intravenous use, 21% endorsed oral use (n = 97), 19% endorsed intranasal use (n = 89), and 16% endorsed using by inhalation (n = 73). All demographic and drug use questions were optional (no forced responding) to allow for greater participant autonomy and choice in which questions were answered. Therefore, while there were 499 valid survey responses, not every question had 499 responses.

The maximum possible endorsement of PBSO in the current survey was 192. On average, participants had a mean score of 105.2 (SD = 30.8) suggesting a frequency of endorsement slightly above “occasional”. See Table 1 for average endorsement of each PBSO item. The maximum possible endorsement for past-month HR service utilization was endorsement of all 11 items. Participants in the current sample endorsed using an average of 2.4 (SD = 2.0) HR strategies in the past-month.

### 3. 1 Psychometric Properties of the PBSO

Cronbach’s alpha for the PBSO in the current sample was 0.95, suggesting high internal consistency. Split half reliability in the current sample was 0.96, also supporting high internal consistency.

### 3.2 Exploratory Factor Analysis (EFA)

Bartlett’s test of sphericity was statistically significant (p < 0.001), suggesting that the data were appropriate for factor analysis. The Kaiser-Meyer-Olkin measure of sampling adequacy (0.92) indicated that the sample size was adequate and appropriate for factor analysis.

In the initial EFA, 26 items had loading < 0.40, while four items demonstrated cross-loading (loadings < 0.32 on more than one factor), and one item did not demonstrate fit with any factor. These five items were dropped, and a second EFA using principal axis extraction and oblimin rotation was used for the remaining 26 items. Five factors were detected with eigenvalue factor loadings greater than or equal to 1.0 (eigenvalues = 10.38, 2.09, 1.69, 1.25, and 1.15). Examination of the scree plot along with parallel analysis supported a 5-factor structure of the PBSO, accounting for 54% of total variance. Examination of correlations between factors suggested a level of association appropriate for oblimin rotation and RMSR (0.02), RMSEA (0.03), and TLI (0.986) all suggested adequacy of the factor model.

Examination of the pattern matrix suggested that ten items mapped onto Factor 1 (accounting for 18% of total variance), seven items mapped onto Factor 2 (11% of total variance), four items mapped onto Factor 3 (10% of total variance), four items mapped onto Factor 4 (10% of total variance), and two factors mapped onto Factor 5 (6% of total variance). The first factor appeared related to health-service seeking or “external” strategies that individuals had to proactively seek out (attending training on OD prevention, fentanyl test strips, accessing wound care, etc.). The second factor appeared related to internally implemented and dose-reduction strategies (sampling a small amount before using more, avoiding mixing drugs, buying a set amount, etc.). The third factor related to social strategies (taking turns when using with others, ensuring use partners have access to and know how to use naloxone, etc.). The fourth factor contained items related to IDU (avoid shared needles, always using new needles, using clean works, disposing of needles safely). Finally, the fifth factor related to testing for HIV and Hepatitis C. See Table 1.

### 3.3 Confirmatory Factor Analysis (CFA)

Following EFA, we conducted a CFA, specifying the five factors suggested by EFA. Results of the CFA indicated that the 5-factor model was a marginal fit for the sample. Generally, indices of good fit include: RMSEA < 0.08, SRMR < 0.08, CFI > 0.90, NFI > 0.90, and NNFI > 0.95 (Tabachnick & Fidell, 2013; Kline, 2015). Though the observed SRMR (0.07) indicated good fit, the RMSEA reflected a marginal fit (0.09), and remaining indices did not demonstrate a good fit. All factor loadings in our model were significant at p < 0.00. Given the poor fit of the model, additional work is needed to validate and confirm a 5-factor structure or identify other factor structures.

### 3.4 Criterion Validity: Association between PBSO and other measured outcomes

Bivariate correlations were used to examine the relationship between PBSO endorsement and other outcomes. Endorsement of PBSO and past-month HR service utilization were moderately correlated (r = .50), and a linear regression suggested a significant association between these variables (F (497, 1) = 145.7, p < .0001, r^2^ = .23). A small but statistically significant negative correlation was observed between PBSO score and days of opioid use per week (r = − .21; p < 0.001), with a significant association (F(368, 1) = 16.26, p < 0.001, r^2^ = .04). Similarly, PBSO score was significantly negatively correlated with self-reported lifetime number of opioid overdoses (r = − 0.20; p < .001), but no significant regression was observed between these variables.

### 3.5 Responses to Open-Ended Question

Following presentation of above PBSO items, participants were presented with an open-ended question that asked, “What are some other strategies that you use to keep yourself safe while using opioids or other drugs?”. One-hundred and forty-four participants provided a response to this question. Responses were coded independently by MG and RL and coding schemes were compared and refined to reach a final coding scheme. Responses were coded based on content and 65% of responses (n = 94/144) noted strategies already captured in the scale. For example, within these responses, participants mentioned buying from one source, ensuring access to naloxone, avoiding mixing opioids with alcohol, use of new needles, using with others rather than alone, etc. Three additional respondents indicated that all PBS strategies were adequately included in the scale. Of the 144 responses, 84 responses were coded as containing more than one theme. Identified themes included: controlling the dose or using only small amounts (n = 44), setting a limit on the amount used or the timeframe of use in a given day (n = 27), use of MOUD (n = 8), only using at home or avoiding use in public places (n = 6), use of disinfectants (n = 9), use in a rescue position (n = 3), avoiding fentanyl (n = 10), altering route of administration to reduce risk (n = 7), use of a pulse oximeter (n = 2), and seeking out knowledge from other individuals using drugs or online communities about the drug supply (n = 11).

## 4. Discussion

The current study sought to develop, validate, and explore the factor structure of a brief, self-report scale measuring PBS in the context of opioid use (PBSO). An initial EFA of the 32-item PBSO developed for the present study suggested a 5-factor structure, with one item that did not load onto any extracted factors, and four items demonstrating cross-loading. These factors were dropped and a second EFA on the remaining 26-items also suggested a 5-factor structure, accounting for 51% of the total variance. Extracted factors appeared to correspond to health-service seeking or extrinsic strategies (Factor 1), individually implemented and dose-reduction strategies (Factor 2), social strategies (Factor 3), and IDU-related strategies (Factor 4). The PBSO scale adapted for the current study demonstrated high internal consistency, and appears to be a reliable measure, though further work should continue to validate this scale in larger, more ethnically and gender-diverse samples. Initial examinations of validity of the PBSO suggest PBSO scores are significantly positively associated with past-month HR service utilization. This suggests that with further validation, the PBSO scale may provide a helpful tool for understanding engagement in protective or buffering strategies for those who use opioids. Further, analyses suggest that PBSO scores may be negatively related to frequency of opioid use and the total number of self-reported opioid overdoses, again providing preliminary evidence for the validity of the PBSO.

### 4.1 Conclusions

As this study was the first to develop and test the PBSO, further work is needed to validate this scale in larger, more diverse samples, and efforts to co-administer the PBSO in settings where objective measurement of opioid use, opioid overdose, and other relevant clinical outcomes is warranted. Though compelling, this work is not without limitations. First, survey respondents include individuals using opioids recreationally, individuals only reporting MOUD use, and individuals reporting MOUD and non-prescribed opioid use. While this represents a wider population of individuals with opioid use, the way questions were asked did not allow us to accurately conduct item variance testing based on opioid use group. Future work incorporating the PBSO into settings where opioid use type can be objectively measured and further categorized would allow for invariance testing, which was not possible in the current sample. Future work exploring whether factor scores differentially relate to relevant health outcomes is also warranted. As prior PBS scales were first developed and implemented in non-clinical settings (Martens et al., 2005), future work must consider PBSO in the context of individuals with OUD or clinically significant problems associated with opioid use.

## Figures and Tables

**Table 1:** Sample Characteristics

	N (%)

**Sex**	

Male	327 (68%)

Female	134 (28%)
Transgender	10 (2%)
Non-Binary	5 (1%)
Other	5 (1%)

**Race**	

African American or Black	59 (12.0%)

White	344 (70%)

Native Hawaiian or Pacific Islander	10 (2.0%)

American Indian or Alaska Native	37 (7.5%)

Asian	23 (4.7%)

Other	5 (1.0%)

**Ethnicity**	

Hispanic or Latino	129 (30.5%)

Non-Hispanic or Latino	294 (69.5%)
**Route of Opioid Administration**	152 (32.9%)
Intravenous	89 (19.26%)
Intranasal/Insufflated	73 (15.8%)
Smoked	97 (21%)
Oral	

	M (SD)

**Age**	31.1 (8.0)

**Opioid Use (days per week)**	3.9 (2.0)
**PBSO Sum**	105.2 (30.8)
**Past-month HR utilization Sum**	2.4 (2.1)

**Table 2:** PBSO Items and EFA Results

	Sample Mean (SD)	% Not Endorsing strategy	Factor 1 loading	Factor 2 loading	Factor 3 loading	Factor 4 loading	Factor 5 loading
2. I use fentanyl test strips	3.2 (1.6)	2.6%	0.44				
10. I attend trainings and meetings focused on overdose prevention	3.0 (1.6)	1.6%	0.59				
11. I attend trainings focused on administering naloxone	3.0 (1.6)	1.8%	0.53				
17. I carry and/or keep a supply of methadone	3.1 (1.7)	2.6%	0.60				
18. I buy methadone instead of heroin/fentanyl if I’m concerned about what’s in the drug supply	3.0 (1.7)	4.0%	0.67				
19. I buy buprenorphine (Suboxone, Subutex) instead of heroin/fentanyl if I’m concerned about what’s in the drug supply	2.9 (1.6)	3.8%	0.62				
25. I use opioids at a safe consumption site / overdose prevention site	2.9 (1.6)	1.2%	0.70				
30. I use PrEP (pre-exposure prophylaxis)	3.0 (1.7)	4.2%	0.93				
31. I use PEP (post-exposure prophylaxis)	2.8 (1.7)	5.0%	0.92				
32. I seek out wound care for any wounds resulting from IV use	3.3 (1.8)	8.4%	0.43				
1. I carry naloxone/Narcan	3.5 (1.6)	0.8%		0.61			
3. I “sample” a small amount before using more	3.7 (1.6)	1.4%		0.51			
14. I avoid mixing opioids and other drugs	3.6 (1.5)	1.2%		−0.67			
16. I carry and/or keep a supply of buprenorphine (Suboxone, Subutex)	3.2 (1.6)	1.6%		0.50			
20. I only buy a set amount of heroin/opioids at one time	3.7 (1.6)	2.2%		0.63			
21. I use opioids intranasally instead of intravenously	3.2 (1.6)	2.8%		0.51			
24. I avoid driving and/or operating heavy machinery while using opioids	3.9 (1.6)	2.2%		−0.49			
4. I use with others rather than alone	3.3 (1.6)	1.0%			0.75		
5. When I’m using with other people, we take turns	3.1 (1.7)	5.2%			0.48		
6. I ensure the people I’m using with have naloxone/Narcan	3.3 (1.7)	3.8%			1.03		
7. I ensure the people I’m using with know how to use naloxone	3.4 (1.7)	3.8%			0.91		
8. I avoid sharing needles with others	4.1 (1.9)	8.6%				0.47	
9. I dispose of used needles at a drop box or designated safe spot	3.5 (2.0)	8.6%				0.54	
12. I only use new needles	3.7 (1.9)	9.2%				0.93	
13. I only use clean works	3.8 (1.9)	8.8%				0.85	
26. I get tested for HIV at least once per year, and more often if I’m concerned about an exposure	3.5 (1.7)	3.0%	0.52				0.78
27. I get tested for Hepatitis C at least once per year, and more often if I’m concerned about an exposure	3.4 (1.7)	3.4%	0.60				0.85
15. I buy heroin/fentanyl from a trusted source*	3.7 (1.7)	4.2%		0.57	0.34		
22. I smoke opioids instead of using them intravenously *	3.1 (1.6)	2.0%	0.39	0.38			
28. I seek out resources/knowledge on ways to prevent transmission of HIV*	3.2 (1.7)	3.6%	0.42				0.52
29. I seek out resources/knowledge on ways to prevent transmission of Hepatitis C	3.2 (1.7)	3.8%	0.51				0.46
23. I only use safe smoking devices (Pyrex pipe stems, rubber mouthpieces)	3.2 (1.8)	7.8%	0.39				

* Items demonstrating cross-loadings or low factor loadings dropped

## Data Availability

The datasets generated and/or analysed during the current study are not publicly available but are available from the corresponding author on reasonable request.
